# Evaluation of data sources and approaches for estimation of influenza‐associated mortality in India

**DOI:** 10.1111/irv.12493

**Published:** 2017-12-02

**Authors:** Venkatesh Vinayak Narayan, Angela Danielle Iuliano, Katherine Roguski, Partha Haldar, Siddhartha Saha, Vishnubhatla Sreenivas, Shashi Kant, Sanjay Zodpey, Chandrakant S. Pandav, Seema Jain, Anand Krishnan

**Affiliations:** ^1^ Centre for Community Medicine All India Institute of Medical Sciences New Delhi India; ^2^ Centers for Disease Control and Prevention Atlanta GA USA; ^3^ Centers for Disease Control and Prevention New Delhi India; ^4^ Indian Institute of Public Health New Delhi India

**Keywords:** India, influenza, mortality

## Abstract

**Background:**

No estimates of influenza‐associated mortality exist for India.

**Objective:**

To evaluate national mortality and viral surveillance data from India for assessing their appropriateness in estimating influenza‐associated mortality using varied analytic approaches.

**Methods:**

We reviewed influenza virus surveillance data from a national influenza surveillance network. We also reviewed national mortality data from Civil Registration System (CRS), Medical Certification of Cause of Death (MCCD) and the Sample Registration System (SRS). We compared and scored the different sources of mortality data using specific criteria, including the process of cause of death assignment, sample size, proportion of ill‐defined deaths, representativeness and availability of time series data. Each of these 5 parameters was scored on a scale from 1 to 5. To evaluate how to generate an influenza‐associated mortality estimate for India, we also reviewed 4 methodologic approaches to assess the appropriateness of their assumptions and requirements for these data sets.

**Results:**

The influenza virus surveillance data included year‐round sample testing for influenza virus and was found to be suitable for influenza mortality estimation modelling. Based on scoring for the 5 mortality data criteria, the SRS data had the highest score with 20 of 25 possible score, whereas MCCD and CRS scored 16 and 12, respectively. The SRS which used verbal autopsy survey methods was determined to be nationally representative and thus adequate for estimating influenza‐associated mortality. Evaluation of the modelling methods demonstrated that Poisson regression, risk difference and mortality multiplier methods could be applied to the Indian setting.

**Conclusion:**

Despite significant challenges, it is possible to estimate influenza‐associated mortality in India.

## 1 INTRODUCTION

Influenza‐associated mortality is difficult to estimate^1^ because few patients with respiratory symptoms who present to the hospital are tested for influenza virus, and death may be related to secondary complications when influenza viruses are no longer detectable. Thus, laboratory‐confirmed influenza deaths are thought to underestimate the true number of deaths caused by influenza. Most influenza‐associated mortality estimations use ecological modelling of viral surveillance and vital records data to assess excess deaths during periods of influenza virus circulation.^2^, ^3^, ^4^ Newer methods utilizing additional parameters, such as influenza virus subtype or measures of temperature and humidity, or alternative statistical techniques, such as the use of splines, to improve estimation are also available.^5^, ^6^

Estimating influenza‐associated mortality in tropical climates and low‐ and middle‐income countries has specific challenges including different seasonal patterns within a country, multiple peaks or year‐round activity, weak influenza virological and epidemiological surveillance systems, and incomplete population‐level vital records data.^3^, ^7^ India, 1 of the most populous and largest tropical climate developing countries, has similar challenges for estimating influenza‐associated mortality.^8^, ^9^, ^10^ India has a population of more than 1.3 billion people and consists of 29 states and 7 Union Territories^1^ (UT).^11^ All‐cause mortality rates are estimated to be 7.1 deaths/1000 persons with the highest rates among children <5 years old (55 deaths/1000) and ≥60 years (44.9 deaths/1000).^12^ While respiratory infections are considered to be 1 of the most common causes of death,^13^ the exact burden of influenza is not known. There are currently no estimates of influenza‐associated mortality for India, and the applicability of published statistical modelling techniques in the Indian context is unclear. A similar set of challenges exists for other developing countries in the region.^14^ However, estimating influenza‐associated mortality is important to understand the impact of influenza virus infection and to estimate the number of deaths among target groups in India. We reviewed the appropriateness of different analytic approaches to estimate influenza‐associated mortality in conjunction with an evaluation of the viral surveillance and mortality data available in India.

## 2 METHODOLOGY

Estimation of influenza‐associated mortality requires quality information about influenza virus circulation and deaths.^4^, ^7^ To develop an approach for estimating influenza‐associated mortality for India, we assessed the availability and quality of influenza virus surveillance and mortality data. Based on earlier work on mortality data evaluation by the World Health Organization (WHO),^15^ which has also been applied to mortality data in India,^10^ we used scoring criteria to assess the quality of mortality data as appropriate for influenza mortality estimation. We then evaluated published modelling methods to estimate influenza‐associated mortality^4^, ^7^ to determine whether these methods could be applied to Indian data.

### 2.1 Influenza virus surveillance data sources

**Figure 1:**
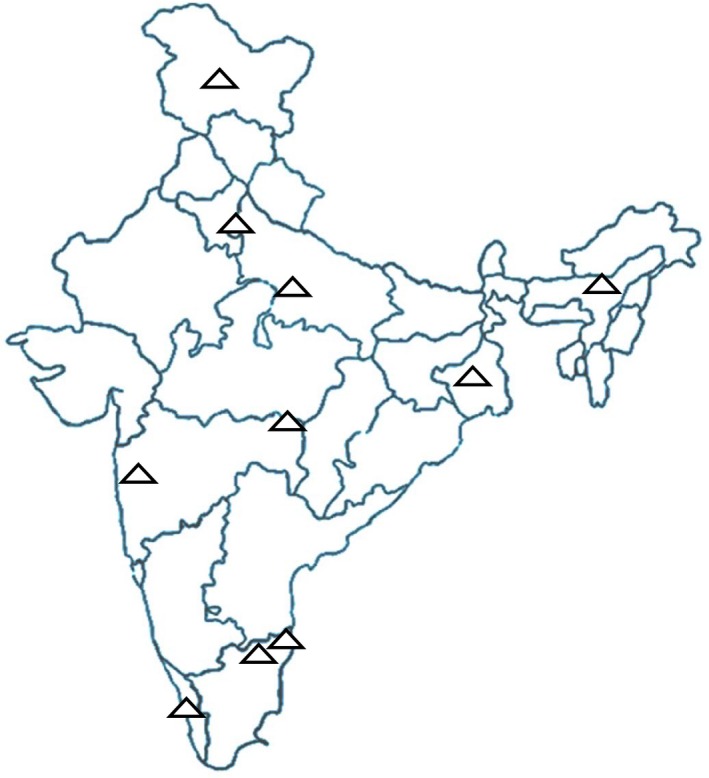
ICMR‐NIV (Indian Council of Medical Research‐National Institute of Virology) Laboratory surveillance network of influenza in India. Δ: Influenza surveillance network laboratory

A laboratory‐based surveillance network for influenza virus detection was established by the Indian Council of Medical Research (ICMR), India, in 2004, with the National Institute of Virology (NIV)^8^, ^16^ (Figure 1). Patients with influenza‐like illness(ILI) presenting at outpatient departments and hospitalized patients with severe acute respiratory infection(SARI) were enrolled randomly into the surveillance system. ILI was defined as sudden onset of fever >38°C or history of fever in the past 3 days, and cough or sore throat or rhinorrhoea.^17^ SARI was defined as an ILI case with difficulty in breathing or clinically suspected pneumonia (in children) with an increased respiratory rate as per the Integrated Management of Childhood Illness definitions.^18^ This surveillance system is the only source of continuous and laboratory‐confirmed seasonal influenza surveillance data in India; thus, we assessed the quality of this data set by evaluating the criteria for specimen collection, influenza testing methods, sample size, proportion of unsubtyped viruses, geographic representativeness and availability of weekly virology data.

In addition to the ICMR‐NIV influenza surveillance system, influenza surveillance is also undertaken by the Integrated Disease Surveillance Programme (IDSP) of the National Centre for Disease Control (NCDC) since 2009 in response to the emergence of influenza A(H1N1)pdm09 virus.^19^ This surveillance system is designed to respond to outbreaks of respiratory infection and is currently only set up to identify infections of influenza A(H1N1)pdmo09 virus. Further, there are no detailed publications publically available to use to evaluate this system, thus it was not included in our analysis.^19^

### 2.2 Mortality data sources

There are 3 available sources of national mortality data in India, including the Civil Registration System (CRS),^20^ Medical Certification of Causes of Death (MCCD)^21^ and Sample Registration System (SRS).^22^ The first data source is the CRS, which is the legal mandatory reporting of all deaths by the next of kin in India. These data are compiled by each state and then collated at the national level.^20^ The second source is the MCCD, which is the medically certified death reporting system, and mainly includes hospital‐based deaths that occur within a state;^21^ in this system, a death certificate is prepared by the attending medical personnel providing cause of death and sent to state MCCD office for cause of death(COD) code assignment by trained staff using the International Classification of Diseases (ICD‐10) system. The third source is the SRS, which is a nationally representative survey of all deaths occurring in >7000 sampling units across India^22^ and uses a standardized verbal autopsy tool from which deaths are then assigned a COD by trained physicians.

The national mortality data sources (CRS, MCCD and SRS) were each evaluated and scored based on 5 attributes to assess sufficiency for influenza mortality estimation.^10^, ^15^ The evaluation was conducted based on published reports for the CRS,^23^ MCCD^24^ and SRS.^22^ The highest possible score for a system was 25, indicating a high‐quality mortality system, and the lowest possible score was 5 indicating a low‐quality data system. The 5 attributes and scoring are described below:

1. *Process of the COD assignment*: Systems utilizing physician COD assignment were scored higher than systems utilizing verbal autopsy,^25^ and scores were given as: 1: reporting of death by family member, 2: verbal autopsy with computer coding, 3: verbal autopsy with physician coding, 4: COD assignment completed by an attending physician and 5: physician coding followed by consistency checks.
2. *Sample size available*: Systems that reported more deaths were scored higher than those that reported fewer deaths. Scores were allocated as 1: ≤5000, 2: 5000‐10 000, 3: 10 000‐100 000, 4: 100 000‐1 million or 5: ≥1 million annual deaths reported.
3. *Proportion of ill‐defined deaths*: Systems which had fewer ill‐defined deaths, that is deaths coded to symptoms, signs, ill‐defined conditions and incompletely recorded deaths were scored higher than those that reported higher proportions of ill‐defined deaths. Scores were allocated as: 1: proportion of ill‐defined deaths >50%, 2: 35%‐49%, 3: 20%‐34%, 4: 10%‐19% and 5: <10%.^26^
4. *Coverage (national representativeness)*: Data sets which covered a larger country‐level population were scored higher than those with less coverage. Scores were allocated as: 1: coverage <30%, 2: coverage 30% to <50%, 3: coverage 50% to < 70%, 4: coverage 70% to < 90% and 5: coverage ≥90%.
5. *Availability of time series COD data*: To capture the full impact of influenza on mortality, models typically use 1 or more categories of coded mortality data: pneumonia and influenza deaths, respiratory deaths, circulatory deaths or all‐cause deaths.^27^ COD data must also be stratified by age group and time (weeks or months) to use time series methods. Scoring was carried out as follows: 1: systems that provided all‐cause deaths by year and age group; 2: systems that provided all‐cause deaths by month and age group; 3: systems that provided COD, specifically respiratory or circulatory deaths, by year and age group; 4: systems that provided COD provided by age group and month; and 5: systems that provided COD by age group and week.

### 2.3 Selection and review of influenza‐associated mortality estimation methods in India

The WHO document “Practical guide for designing and conducting influenza disease burden studies”^7^ was reviewed to identify studies and methods for influenza‐associated mortality estimation. Analytic methods from this document were studied, and the 23 references regarding these methods from this document were reviewed. Additional PubMed searches to identify methods used to calculate population‐level influenza‐associated mortality estimates were conducted to identify articles from countries with tropical climates. These methods were studied and evaluated to understand data requirements, model assumptions, strengths, limitations and applicability to Indian data.

## 3 RESULTS

### 3.1 Viral surveillance data

For the ICMR‐NIV influenza surveillance network, specimens were tested for influenza virus using standard protocols coordinated by NIV, Pune, WHO National Influenza Center for India. The clinical virology laboratories were located across ten cities in 8 states providing geographical and climatic representation of India (Figure 1). Five to 10 nasopharyngeal swabs were collected each week from participating centres from 2004 to 2013 year‐round. A total of 58 055 samples were tested of which 6810 (11.7%) were found to be positive for influenza.^8^, ^16^ Fewer than 1% of specimens were not classified by influenza type and subtype. Annual compiled estimates of circulating influenza type, subtype and per cent positive have been published,^8^, ^16^ and information is regularly reported to WHO FluNet.^28^ Detailed weekly virological data were obtained through a request to NIV.

### 3.2 Mortality data

After scoring each system (Table 1), the SRS had the highest score with 20 of 25, whereas MCCD and CRS scored 16 and 12, respectively.

**Table 1:** Criteria and scoring methods for evaluating mortality data sources of the Civil Registration System (CRS), Medical Certification of Causes of Death (MCC) and Sample Registration System (SRS)

Criteria and scoring	CRS^23^	MCCD^24^	SRS^22^
Process of cause of death (COD) assignment By attending physician followed by: major quality checks = 5; few/no quality checks = 4; verbal autopsy with physician coding = 3; computer coding = 2; and lay reporting = 1	1 (Lay reporting)	4 (Attending physician‐assigned COD)	3 (Standardized verbal autopsy)
Sample size (No of deaths reported annually) >2 million:5; 200 000‐2 million:4; 20 000 to <200 000:3; 10 000‐20 000 deaths:2; and <10 000 deaths:1	5 (6.1 million deaths [2013])	4 (0.93 million deaths [2013])	3 (0.18 million deaths [2010‐2013])
Proportion of ill‐defined deaths <10% = 5; 10‐19% = 4; 20%‐34% = 3; 35%‐49% = 2; and >50% = 1	1 (Lay reporting)	4 (13.3% [varies by state])	4 (12.4%)
National coverage (representativeness): proportion of deaths captured at national level (>90%: 5; 70%‐90%:4; 50%<70%:3; 30%‐<50%: 2; and <30%: 1)	4 (National: 70.9%)	1 (National: 20.1%)	5 (Nationally representative survey)
Availability of time series COD data( )by cause, week and age group: 5; by cause, month and age group: 4; by cause, year and age group: 3; all cause by month and age group:2; and all cause by year and age group: 1	1 (all cause by year and age group)	3 (by cause, year and age group)	5 (by cause, week and age group)
Total (of 20)	12	16	20

MCCD, Comprises of deaths in hospital; reported by medical personnel; SRS, Systematic survey of 0.6% of deaths; verbal autopsy used; CRS, Comprises of all deaths; reported by next of kin.

The overall score for the CRS system was 12 of 25 (Table 1). The CRS reports all deaths registered within a state. While this is a large number of reported deaths, and CRS received a score of 5 for its sample size, CRS does not assign a specific COD; thus, it received a score of 1 for the COD assignment process. The national coverage in 2013 was 70.9%, leading to a score of 4 on this parameter.^23^ There were marked differences in coverage between states, however, ranging from 100% in 11 states/UTs to <60% in 9 other states/UTs. While state‐specific coverage has improved over the years, there may be an over‐representation, due to instances when people die in cities or states where they are not residents, and their death is therefore reported in the city or state where the death occurred and not their city or state of residence.^29^

The overall score for the MCCD system was 16 of 25 (Table 1). The MCCD system reports medically certified deaths with physician‐assigned COD receiving a score of 4 for process of COD assignment. The 2013 MCCD reported national coverage based on data reported from 31 (from total of 36) states/UT's was 20.1%, thus receiving a score of 2 for coverage.^24^ State variation was also observed such that only 5 smaller states/UTs had coverage >70%, 13 states/UTs had coverage ranging from 20 to 70%, and coverage for the remaining 13 states was <20%. The states prepare aggregated data because MCCD is a state‐based system; these data are further aggregated for the national MCCD report by ICD‐10 death categories stratified by age group. One limitation of the MCCD system is that it is largely implemented in urban hospitals within the state. As a result, the urban MCCD coverage, especially for capital cities, is much higher than coverage in rural areas.^24^

The overall score for the SRS system was 20 of 25 (Table 1). The SRS mortality data were obtained through a continuous nationally representative sample survey of deaths, which utilizes standardized verbal autopsy and ICD‐10 codes that are assigned by trained physicians, which gave it a score of 3 for COD assignment process.^22^ A nationally representative sample of 7 million people (0.6% of India population) was covered through 7597 geographically representative sampling units selected by stratified simple random sampling (including 4433 rural and 3164 urban) across all states of India, thus it received a score of 5 for coverage/national representativeness. Approximately 45 000 deaths are reported annually. Aggregate reports from 2001 to 2003, 2004 to 2006, 2007 to 2009 and 2010 to 2013 are publically available.^22^

### 3.3 Review of published studies for identifying methods for influenza‐associated mortality estimations

Thirteen articles were identified that described methods for estimating influenza‐associated mortality for countries with tropical climates (Table 2).^30^, ^31^, ^32^, ^33^, ^34^, ^35^, ^36^, ^37^, ^38^, ^39^, ^40^, ^41^, ^42^ Four different methods for influenza‐associated mortality were identified and evaluated: Poisson regression methods,^31^, ^32^, ^33^, ^35^, ^36^, ^37^, ^38^, ^40^, ^41^, ^42^ linear regression using a Serfling approach,^31^, ^39^ risk difference method^35^ and multiplier methods.^30^, ^34^, ^39^ Data requirements and strengths and limitations were reviewed (Table 3).

**Table 2:** List of studies reviewed to identify methods available for influenza mortality estimation in tropical countries^a^

	Country (Alphabetical order)	Method (s) utilized	First author and publication year
1.	Bangladesh	Mortality multiplier	Homaira 2012^30^
2.	China	Negative binomial regression Linear regression with Serfling methods	Feng 2012^31^
3.	China southern	Negative binomial regression	Wang 2014^32^
4.	China south, Hong Kong & Singapore	Poisson regression	Yang 2011^33^
5.	Costa Rica	Mortality Multiplier	Saborio 2014^34^
6.	Hong Kong	Poisson regression Rate difference	Wong 2004^35^
7.	Hong Kong	Regression correlation model	Li 2006^36^
8.	Hong Kong	Poisson regression	Yang 2012^37^
9.	Hong Kong	Linear regression	Wu 2012^38^
10.	Latin America (PAHO)	Linear regression with Serfling methods Mortality multiplier	Cheng 2015^39^
11.	Singapore	Negative binomial regression	Chow 2006^40^
12.	Thailand	Bayesian regression	Cooper 2015^41^
13.	Thailand	Negative binomial regression	Aungkulanon 2015^42^

a [Obtained through PubMed search, using influenza [Title] AND mortality [Title] OR influenza [Title] AND deaths [Title]].

**Table 3:** Review of the data requirements, strengths and limitations of analytic methods available for influenza mortality estimation

Method	Data requirements	Strengths	Limitations
Poisson Regression Method and derivatives^3^, ^4^, ^7^	Influenza surveillance data: weekly or monthly (by subtypes) Mortality data: weekly or monthly	Produces estimate of numbers and rates of deaths by influenza type and subtype Accounts for changes in population size over time Also able to incorporate other variables, such as circulation of other pathogens (eg respiratory syncytial virus); and climatic variables (eg temperature, humidity) thereby accounting for confounding	Requires consistent, robust viral surveillance data and at least 5 years of mortality data for stable estimates by type and subtype
Serfling Regression Method^2^, ^39^, ^47^	Well‐defined influenza seasonality Viral surveillance data not required Mortality data: weekly or monthly	Robust viral surveillance data not required Useful for temperate countries with clear seasonal patterns of influenza and relatively easy to apply	At least 5 years of mortality data required Not applicable for subtropical and tropical countries lacking well‐defined influenza seasonality
Risk Difference Method^3^, ^35^	Influenza surveillance data: weekly or monthly Mortality data: weekly or monthly	Can be used in countries with varied influenza seasonality and less than 5 seasons of data Does not require manual definition of epidemic thresholds Allows other factors (eg circulation of other viruses) to be incorporated	Mortality estimates differ with epidemic threshold Cannot estimate influenza type/subtype‐specific mortality Does not account for other seasonal factors such as temperature or humidity
Multiplier model approach^30^, ^34^, ^39^	Influenza surveillance data: monthly/annual by age group Mortality data: monthly/annual by age group	Novel approach adopted in the absence of viral surveillance data of at least few years Approach for countries that do not have strong vital records or autopsy data	Data for more years needed to yield more representative estimates Requires data on respiratory, influenza‐like illness (ILI) or pneumonia and influenza (P & I) mortality

### 3.4 Applicability of influenza‐associated mortality estimation methods in India

Although data about both influenza virus circulation and mortality for the same period of time are needed to estimate influenza‐associated mortality, each estimation method is based on certain model assumptions, and specific data requirements may differ. For a large country, such as India, state‐level or regional calculations of influenza‐associated death may be needed given the differences in virus circulation in the country.^16^

#### 3.4.1 Poisson regression method (generalized linear equations with viral surveillance data)

This method is frequently used when robust influenza surveillance data by type and/or subtype with 5 or more years of data are available.^3^, ^4^, ^7^ The mortality data should be representative of at least 70%‐90% of all deaths to provide stable estimates of mortality from regression models.^26^ Additionally, to portray reasonable quality for ICD‐coded death data, the proportion of ill‐defined deaths should be <20% for obtaining reliable estimates.^26^ This method, including its negative binomial variant, is commonly used by studies on influenza mortality estimation and can provide results by virus type and subtype.^31^, ^32^, ^33^, ^35^, ^36^, ^37^, ^38^, ^40^, ^41^, ^42^ Influenza surveillance data of NIV‐ICMR network from India provide data by type and subtype at the national level, and thus, these methods could be applied to this data set. However, the Poisson approach could only be considered for the SRS mortality data set since for CRS and MCCD, the coverage varies widely among states and thus renders them unsuitable for incorporation into regression models.

#### 3.4.2 Linear regression using Serfling methods

This method and its derivatives require well‐defined seasonality data and are applicable mostly to temperate climate regions where there is typically 1 major peak of influenza virus circulation activity each year. Mortality is estimated with this method by attributing all‐excess mortality to influenza during the influenza virus circulation epidemic period as defined by viral surveillance data or other methods.^2^, ^39^ As a substantive number of cases are identified throughout the year and the epidemic periods are difficult to define, the Serfling approach might not be an appropriate method for data from India.^8^, ^16^

#### 3.4.3 Risk difference method

This method relies on the definition of epidemic and non‐epidemic (assumed to have low or no influenza viruses in the circulation as a baseline or control) periods so that the difference in observed mortality between the 2 periods can be assessed to estimate the excess impact. The epidemic cut‐off is arbitrarily decided based on percentage of influenza specimens testing positive (eg 10% or 15%).^3^ Calculation of the excess mortality is made by subtracting the number of deaths during the non‐epidemic periods from the observed numbers of deaths during influenza epidemic periods. This method is more flexible in terms of data requirements and has been used with <5 years^35^ of data. This method can be attempted for Indian states and sites where there is good coverage (>70%) of mortality data either monthly or weekly for all‐cause, respiratory or circulatory deaths.

#### 3.4.4 Multiplier approach

This method requires age‐specific number of ICD‐coded deaths for specific causes such as pneumonia and influenza (P & I) deaths to multiply the proportion of influenza per cent positive for that age group to calculate estimates of influenza‐associated P & I mortality. This can be done for areas with adequate (>70%) coverage of deaths available by month^30^ or year.^34^ Although the multiplier method does not provide information on type and subtype‐specific mortality, the data requirements are quite flexible in comparison with regression techniques. This model additionally assumes that after adjusting for age and week, a similar per cent of survivors and decedents would have tested positive for influenza if all persons had been sampled. Cheng et al^39^ used the proportion of excess influenza deaths to all respiratory deaths to generate an attributable fraction of influenza‐associated deaths in certain Pan American Health Organization (PAHO) countries. This was then applied to other PAHO countries that had adequate coverage of mortality data but no viral surveillance data. Although viral surveillance data are available for India, the CRS and MCCD national data sets have inadequate mortality data coverage for application of this method. While the SRS national data set can be used with this methodology, it could also be attempted with subnational data sets having adequate coverage.

## 4 DISCUSSION

Through our systematic evaluation of data sources and methods for estimating influenza‐associated mortality in India, we identified that robust influenza virus surveillance data were available from a national influenza surveillance network and could be applied to several different methods. Among the national mortality data sources evaluated, SRS had the highest score and therefore is the most appropriate for inclusion in models for excess influenza‐associated mortality estimation. Except for the Serfling method, which requires very well‐defined influenza virus seasonality, the methods that we reviewed (Poisson regression, rate difference and multiplier methods) could all be applied to Indian settings.

Earlier reviews of the SRS database also demonstrated it to be the most representative source for mortality in India,^29^, ^43^ and it has been extensively utilized to obtain disease burden estimates for India for conditions other than influenza.^13^, ^44^, ^45^ Adoption of a uniform standardized verbal autopsy tool by trained investigators followed by systematic ICD‐10 coding assigned independently by 2 trained physicians has ensured the quality of the COD data from the SRS.^46^ As the routine reporting systems of CRS and MCCD provide inadequate coverage at the national or regional level, the survey data of SRS are widely utilized because of its coverage, data quality and ability to obtain regional data. However, as CRS and MCCD are state‐based systems with wide variation in coverage across states, further evaluation is needed to determine which states may have higher quality data available. In states with the most robust data, both CRS and MCCD data could help to focus efforts to gather data for generating state‐specific estimates as health is a state issue in India and states could apply resources accordingly.

State or regional‐level estimation is also important because influenza virus circulation patterns may vary within India. While majority of the country experience influenza virus circulation during the monsoon season, a few northern states with more temperate climates experience peaks in influenza activity during winter months.^16^ As the timing of the monsoon season and the winter months vary between the states, the ideal method to estimate mortality in India if all necessary data were available would be to calculate state or regional‐level estimates by age for those areas with similar virus circulation. Depending on population represented in these estimates, methods can be developed to generate national estimates or previously described methods could be considered.^39^ The CRS data in states with >70% CRS coverage could be analysed to estimate influenza‐associated all‐cause excess deaths to generate state‐specific estimates, although it may overestimate deaths due to the inclusion of unrelated death categories such as poisoning and accidents.^47^ Cities within states with good MCCD coverage (>70%) could also be analysed using COD data with corresponding regional virology data to estimate influenza‐associated deaths for these cities; similar analyses have been performed for 8 cities in China.^31^ Also, SRS data could be obtained for states or group of states and analysed accordingly.

As weekly virological data from NIV provided influenza virus data by subtypes and coincided with weekly SRS data of sufficient sample size at the national level, the Poisson regression was determined to be the most appropriate method for deriving national influenza‐associated mortality estimates in India. In the future, for subnational mortality data which may become available but may not meet data requirements for regression models, the use of both the multiplier and rate difference methods could be explored for Indian estimations. In recent years, while the multiplier method is increasingly being used in countries without available detailed time series data,^30^, ^34^, ^39^ only a few studies have used the risk difference method to calculate an influenza mortality estimation.^3^, ^35^

There are some limitations to this review of data and approaches for influenza mortality estimation. As this evaluation was based mainly on data review, carrying out the actual estimation could present with additional issues pertaining to application of the estimation methods to Indian data. Also, we were only able to evaluate national influenza surveillance data that were available either publically or by request. Thus, there may be influenza virological data that we did not have access to but which could be added or utilized at a local level. Similarly, we evaluated mortality data sets that were available either publically or by request. Both CRS and MCCD data may have valuable data at the state and local level which could contribute to these efforts at subnational level.

## 5 CONCLUSIONS

Although there are considerable challenges for estimating influenza‐associated deaths in India, data sources are available that would allow use of modelling methods for this estimation. This evaluation of data sources and analytic methods provides ways to use the data within its limitations to provide information on influenza burden that could inform public health policy. Because of India's size and global importance, modelling methods may be a highly relevant approach to defining public health priorities and strategies. Thus efforts to retain and grow both the virology and mortality data sources in India would help to work towards these goals.

## DISCLAIMER

The findings and conclusions in this report are those of the authors and do not necessarily represent the official position of All India Institute of Medical Sciences, New Delhi, and US Centers for Disease Control and Prevention, Atlanta.

## Note

1 Type of administrative division in India: too small to be a state, population is lesser (<20 million), usually ruled by Union/central Government.
